# An Anthracene-Based Bis-Stilbene Derivative as Luminescent Materials for Organic Light Emitting Diodes

**DOI:** 10.3390/ma16103685

**Published:** 2023-05-12

**Authors:** Hui Wang, Houlin Wu, Guangling Bian, Ling Song

**Affiliations:** 1 College of Chemistry and Materials Science, Fujian Normal University, Fuzhou 350007, China; wanghui@fjirsm.ac.cn; 2 Fujian Science & Technology Innovation Laboratory for Optoelectronic Information of China, Fuzhou 350108, China; 3 The Key Laboratory of Coal to Ethylene Glycol and Its Related Technology, Fujian Institute of Research on the Structure of Matter, Chinese Academy of Sciences, Fuzhou 350002, China; wuhoulin@fjirsm.ac.cn; 4 Fujian College, University of Chinese Academy of Sciences, Fuzhou 350002, China

**Keywords:** organic fluorescence materials, anthracene, OLEDs, organic semiconductors

## Abstract

In this work, a new luminescent material of a small-molecule stilbene derivative (BABCz) containing anthracene was designed and synthesized by three simple reactions. The material was characterized by ^1^H-NMR, FTMS, and X-ray and tested using TGA, DSC, UV/Vis, fluorescence spectroscopy, and atomic force microscopy. The results demonstrate that BABCz has luminescence properties with good thermal stability and can be doped with 4,4′-bis(N-carbazolyl)-1,1′-biphenyl (CBP) to prepare highly uniform films, which allows the fabrication of OLED devices with ITO/Cs_2_CO_3_:BABCz/CBP:BABCz/MoO_3_/Al configuration. This simplest device in the sandwich structure emits green light at 6.6–12 V and has a brightness of 2300 cd/m^2^, indicating the potential of this material in OLED manufacturing.

## 1. Introduction

Organic semiconductors have been extensively studied in recent decades due to their excellent optical and electrical properties, wide material sources, easy modification and regulation, light weight, high mechanical flexibility, simple manufacturing process, low production cost, and other advantages. They have great application potential in many fields, including organic light-emitting diodes (OLED) [1,2,3], organic light emitting transistors (OFET) [4,5,6], and organic solar cells (OSC) [7,8]. Organic electroluminescent materials are a very important class of organic semiconductor materials, which are mainly used in fields such as OLEDs, organic light-emitting transistors (OLETs), and lasers for organic electric pumps. Excellent electroluminescent materials need to exhibit high stability, high carrier mobility, and high solid-state luminescence efficiency simultaneously. However, this class of materials is not easily accessible because high carrier mobility usually requires large and strong conjugated systems and abundant π-π intermolecular interactions to produce good charge transport properties, but organic molecules with such structures usually severely quench solid-state luminescence. Few molecular systems are compatible with good charge transfer and strong solid-state fluorescence emission [9]. To overcome this contradiction, strategies such as host-guest doping [10,11], designing star shaped molecules [12,13,14,15], aggregation-induced luminescence (AIE) molecules [16,17], or thermally activated delayed fluorescence molecules [18,19] were developed and achieved good results.

**Figure 1:**
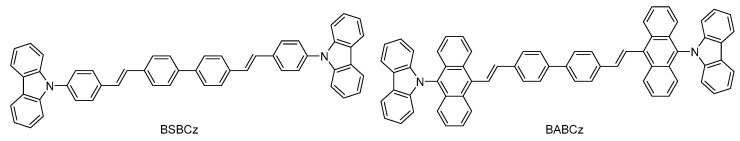
Structures of BSBCz and BABCz.

Among them, 4,4′-bis[(N-carbazole)styryl]biphenyl (BSBCz) is an excellent trans-stilbene fluorescent material and shows important application potential in electro-pumped laser (Figure 1) [20,21]. However, there are some problems, such as solid-state fluorescence quenching and poor stability [22]. In 2018, Adachi et al. introduced alkyl groups into BSBCz, which significantly improved its solubility, and the introduction of cyano groups improved its electron-accepting properties [23]. In 2020, they developed new furyl derivatives to improve their thermal and photostability [22]. In the same year, Turnbull et al., by replacing the biphenyl in BSBCz with fluorene, showed that this modification significantly improved the solubility, molar extinction coefficient, and rate of radiation decay of the material [24]. In summary, the modification of the star molecule BSBCz is still minimal, and robust organic semiconductors with high performance are highly desired.

Anthracene, the smallest polybenzoic fused ring compound, exhibiting a high fluorescence and quantum yield, good solubility, and stability, has emerged as a promising high-performance organic light-emitting semiconductor [25,26,27,28]. As early as the 1960s, electroluminescence phenomena were found in anthracene single crystals. In 1974, the first organic solid-state laser with pure anthracene crystals as the gain medium was reported [29]. Henceforth, anthracene has become a star building block in OLED, OFET, fluorescent probes, organic laser display (OLSD), and electronic materials [30,31,32].

We propose that anthracene, a star skeleton, can be used to adjust the energy level, molecular stacking mode, and stability of BSBCz by utilizing its π-conjugated system and photoelectric properties. Thence, a new anthracene derivative, 4,4′-bis [(N-carbazole) anthracylic-vinyl] biphenyl (BABCz), was designed and synthesized in this work (Figure 1). This new material exhibits good thermal stability and electroluminescent properties. With this material as the light-emitting layer, the simplest three-layer OLED device was successfully developed.

## 2. Methodology and Experimental

### 2.1. Synthesis

#### 2.1.1. Synthesis of 10-Bromoanthracene-9-carbaldehyde

The 9-Anthracene formaldehyde (4.0 g, 19.4 mmol) is dissolved in 90 mL of dry dichloromethane. Under the protection of nitrogen, a dichloromethane (10 mL) solution of liquid bromine (3.728 g, 23.3 mmol) was slowly added at room temperature. After a 30-min reaction, the temperature is raised to 40 °C and refluxed overnight. The reaction solution was slowly cooled to 0 °C, and then the precipitated crude product was filtered and washed with ethanol. After purification by silica gel column chromatography (dichloromethane leaching), 3.5 g of yellow solid product was obtained, with a yield of 63%. ^1^H NMR (400 MHz, CDCl_3_): δ = 11.46(s, 1H), δ = 8.86(d, *J* = 9.37 Hz, 2H), δ = 8.64(d, *J* = 8.42 Hz, 2H), δ = 7.69 − 7.63(m, 4H). ^13^C NMR (100 MHz, CDCl_3_): δ = 193.3, 131.9, 131.8, 130.2, 129.0, 128.9, 127.4, 125.6, 123.8. HRMS (ESI-TOF Positive Ion Mode) *m*/*z* (%): calcd for C_15_H_10_BrO [M+H]^+^ 284.9910, found 284.9914. (NMR and MS spectra are in Supporting Files).

#### 2.1.2. Synthesis of 10-(9H-Carbazol-9-yl)anthracene-9-carbaldehyde

The 10-Bromanthracene-9-formaldehyde (0.5 g, 1.75 mmol), carbazole (0.44 g, 2.63 mol), o-phenanthroline (0.063 g, 0.35 mmol), cuprous iodide (0.66 g, 0.35 mmol), and potassium tert-butoxide (0.39 g, 3.51 mmol) were added to 30 mL of anhydrous toluene, and reacted for 48 h at 110 °C under nitrogen protection. The reaction solution was cooled to room temperature, poured into 50 mL of ice water, and then extracted twice with ethyl acetate. The organic phase was dried by anhydrous sodium sulfate and concentrated to obtain the crude product. The crude product was purified by silica gel column chromatography (carbon tetrachloride/dichloromethane = 10/1 elution) to obtain 0.38 g of bright yellow product with a yield of 58%. ^1^H NMR (400 MHz, CDCl_3_): δ = 11.67(s, 1H), δ = 9.05(d, *J* = 8.99 Hz, 2H), δ = 8.30(d, *J* = 7.55 Hz, 2H), δ = 7.72 − 7.67(m, 2H), δ = 7.38 − 7.28(m, 8H), δ = 6.72 (d, *J* = 7.10 Hz, 2H). ^13^C NMR (100 MHz, CDCl_3_): δ = 193.3, 142.5, 135.9, 132.3, 129.7, 129.2, 127.3, 126.9, 126.4, 124.5, 124.1, 123.3, 120.6, 120.4, 110.2. HRMS (ESI-TOF Positive Ion Mode) *m*/*z* (%): calcd for C_27_H_18_NO[M+H]^+^ 372.1383, found 372.1388. (NMR and MS spectra are in Supporting Files).

#### 2.1.3. Synthesis of 4,4′-bis[(N-carbazole)anthracylic-vinyl]biphenyl (BABCz)

The 10-(9H-carbazol-9-yl)anthracene-9-carbaldehyde (0.5 g, 1.35 mmol) and tetraethyl ([1,1′-biphenyl]-4,4′-diylbis(methylene))bis(phosphonate) (0.245 g, 0.54 mmol) were added to 40 mL of dioxane, and potassium tert-butoxide (0.302 g, 2.7 mmol) was added in batches, and reacted at room temperature for 5 h, then heated to 80 °C for 36 h. The reaction solution was concentrated to dryness under reduced pressure, and the residue was successively washed with methanol and water before recrystallization using dichlorobenzene to afford the yellow product in 0.12 g with a yield of 27%. ^1^H NMR (400 MHz, CDCl_3_): δ = 8.56(d, *J* = 8.87 Hz, 4H), δ = 8.31(d, *J* = 7.40 Hz, 4H), δ = 8.13(d, *J* = 16.58 Hz, 2H), δ = 7.87(q, *J* = 7.97 Hz, 8H), δ = 7.52(t, 4H), δ = 7.38 − 7.25(m, 16H), δ = 7.17(d, *J* = 16.61 Hz, 2H), δ = 6.79(d, *J* = 7.67 Hz, 4H). HRMS (AP-MALDI Positive Ion Mode) *m*/*z* (%): calcd for C_68_H_44_N_2_ [M+H]^+^ 888.3499, found 888.3493. (NMR and MS spectra are in Supporting Files).

### 2.2. Single Crystal Structure of BABCz

An amount of 200 mg of BABCz was dispersed into 20 mL of o-dichlorobenzene, heated to 180 °C for 3 h, and then slowly decreased to room temperature at a rate of 10 °C/h to precipitate single-crystal crystals. A Single Crystal X-ray was performed by the Synergy-R-Mo X-ray diffraction instrument (Rigaku; Tokyo; Japan).

### 2.3. Preparation of BABCz Films

The films were prepared on quartz substrates. The quartz substrate was ultrasonically cleaned by deionized water, acetone, and isopropanol for 15 min, respectively. After the substrate was treated with ultraviolet ozone for 20 min, BABCz pure film and doped film were prepared by vacuum evaporation method (VZZ-400, Beijing Micro-Nano Vacuum Technology Co., Ltd., Beijing, China). 120 nm BABCz pure film was prepared at a deposition rate of 0.6 Å/s. In the doped film, the deposition rate of BABCz is 0.2 Å/s, that of CBP is 1 Å/s, and the thickness of doped film is 120 nm.

### 2.4. OLED Device Fabrication

Commercially available ITO glass substrates were ultrasonically cleaned with deionized water, acetone, and isopropanol for 15 min, respectively. Then, the substrate was treated with UV ozone for 20 min. All materials were vaporized by vacuum thermal evaporation. The device was made according to the following structure: ITO/Cs_2_CO_3_:BABCz (1:6) (35 nm)/CBP:BABCz (5:1) (100 nm)/MoO_3_ (5 nm)/Al (250 nm). The evaporation rates of Cs_2_CO_3_, BABCz, CBP, BABCz, MoO_3_, and Al were 0.1 Å/s, 0.6 Å/s, 1.0 Å/s, 0.2 Å/s, 0.1 Å/s, and 2.0 Å/s, respectively. The film thickness of each layer was monitored by a quartz oscillator.

## 3. Results and Discussion

### 3.1. Synthesis and Structural Analysis

**Scheme 1:**
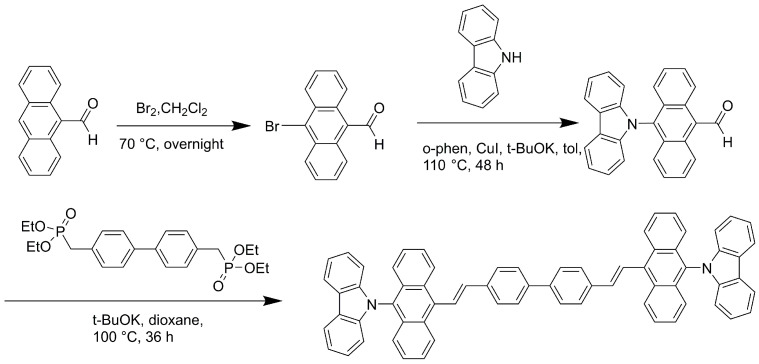
Synthesis of BABCz.

**Figure 2:**
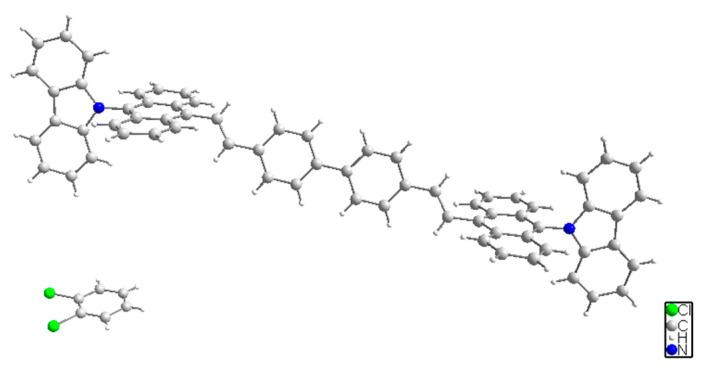
A single crystal structure of BABCz (containing a molecule of dichlorobenzene).

BABCz was synthesized from 9-anthracene formaldehyde by bromination, Buchwald Hartwig cross coupling, and Wittig Horner reactions (Scheme 1). Its structure was confirmed by X-ray diffraction analysis (Figure 2, CCDC: 2246740).

**Figure 3:**
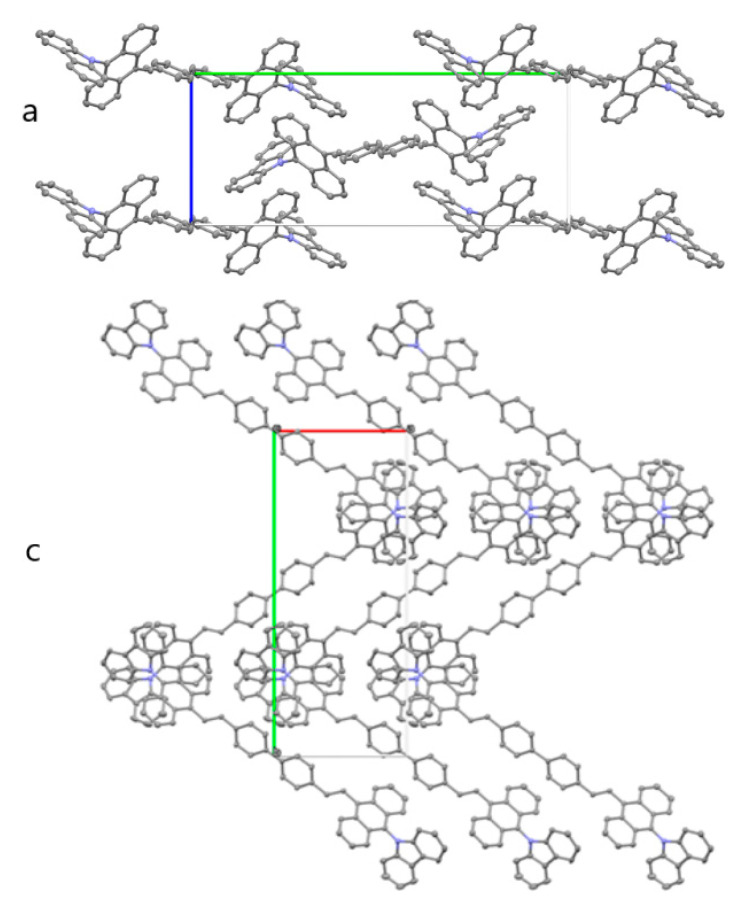
Molecular packing diagram in the single crystal structure of BABCz (Red line for a-axis, green line for b-axis and blue line for c-axis).

As can be seen from the single crystal in Figure 2 and Figure 3, the anthracene is nearly perpendicular to the intramolecular carbazole and styrene building blocks, the two molecules of anthracene are nearly parallel, and the intermediate 4,4′-divinylbiphenyl is perfectly coplanar. In the a-axis direction, intermolecular staggered vertical stacking occurs, and the anthracene of the middle molecule is perpendicular to the carbazole and phenyl rings of the neighboring molecules, respectively. In the c-axis, layered parallel stacking occurs between molecules, and in the b-axis, zigzag stacking occurs between molecules, which is the smallest mode of stacking with conjugation between molecules (σ_H_-π). This unique structure minimizes the fluorescence quenching caused by solid-state stacking.

### 3.2. Performance Testing

**Figure 4:**
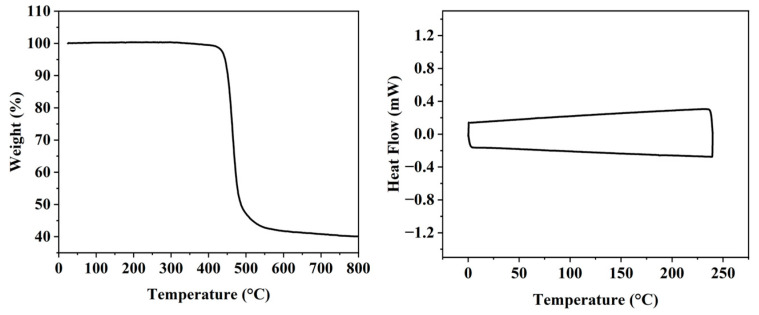
TGA curve (**left**) and DSC curve (**right**) of BABCz.

First, BABCz was analyzed by thermogravimetric analysis with a decomposition temperature (TD, 5% weight loss) of 445 °C (Figure 4). Differential scanning calorimetry (DSC) tests were performed on BABCz and revealed that no significant glass transition temperature (TG) was observed in the range 0–240 °C, indicating good thermal stability of BABCz.

**Figure 5:**
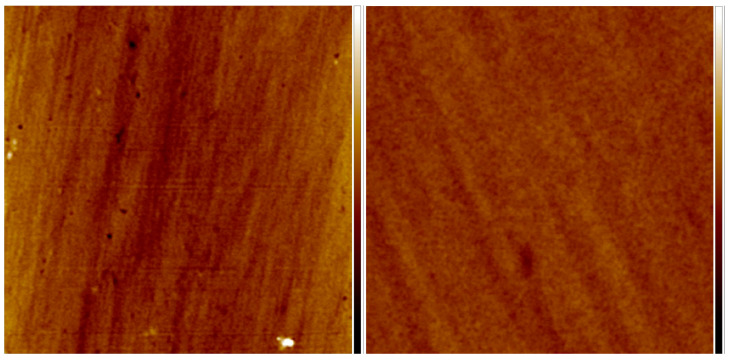
Vacuum-deposited pure film of BABCz (**left**); vacuum-deposited doped film of BABCz/CBP (**right**).

Then, pure films (120 nm) and CBP-doped vacuum-deposited films (120 nm, BABCz/CBP = 1/5) of BABCz were prepared for examining the film-forming properties of materials and the photophysical properties of solid-state films. From the atomic force microscopy (AFM) images of the two films (5 × 5 μm) (Figure 5), it can be seen that the root mean square roughness (RMS) of the doped films is much smaller, only 0.49 nm. The RMS of pure membranes is relatively high, reaching 1 nm. It can be seen that the film-forming performance of this material is not good enough. It is more appropriate to select BABCz and CBP doped film for device preparation.

**Figure 6:**
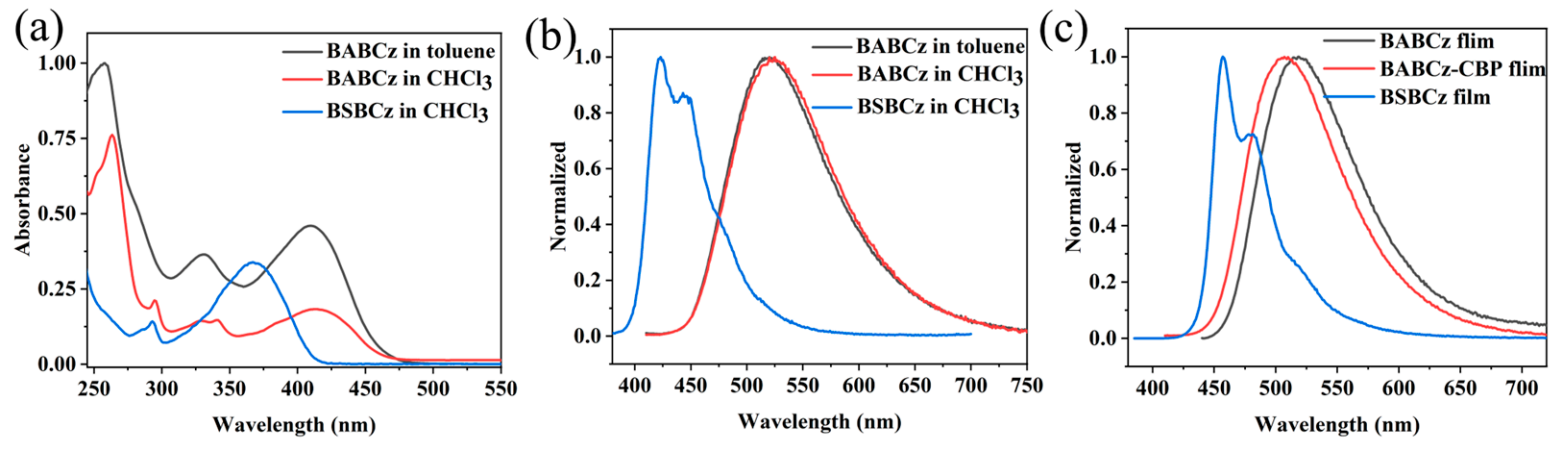
(**a**) UV-visible absorption spectra; (**b**) fluorescence emission spectra in solutions; and (**c**) fluorescence emission spectra in films.

Figure 6 shows the UV-visible absorption spectra and fluorescence emission spectra of BABCz and BSBCz in solutions (5 μmol/L) and films. It can be seen from the figure that the absorption spectra and emission spectra of BABCz relative to BSBCz have different degrees of redshift, which is due to the replacement of the benzene ring by the anthracene ring with a larger π-π conjugate system, which reduces the energy required for electronic transition. Toluene and chloroform have little effect on the fluorescence emission of BABCz. Contrasting Figure 6b,c, it was seen that the emission peaks of BSBCz in vacuum-deposited film were 457 nm and 481 nm, which were red-shifted by about 35 nm relative to that in solution, while for BABCz material from solution to vacuum-deposited film, the emission peaks were all around 520 nm, indicating that the introduction of the anthracene group made the rigidity of the molecule stronger, the force between fractions weaker, and the intermolecular packing mode has less influence on its fluorescence emission. Affected by CBP with a shorter emission wavelength (405 nm), the emission peak was blue-shifted by 11 nm in doped films of BABCz and CBP. The photoluminescence quantum yield (PLQY) of BABCz in chloroform solution was 8.93% (Table 1), which was much lower than that of BSBCz, probably due to the large steric hindrance of the planar structure of anthracycline, which is almost completely perpendicular to the styrene, thus affecting the conjugation delocalization of electrons between the two, which decreased the luminescence efficiency. No effective PLQY was observed in the pure film of BABCz, which may be due to the low luminescence efficiency of the material itself and the poor film quality of the pure films. It is gratifying that the PLQY is significantly improved to 23.33% in the doped films of BABCz and CBP. The fluorescence lifetime of BABCz is 1.42 ns in solution and 1.76 ns in vacuum-deposited doped film with CBP, which is similar to BSBCz, whereas the radiative decay rate constant (k_r_) of the BABCz is significantly smaller than that of the BSBCz, which is detrimental to the material’s amplified spontaneous emission (ASE) performance. Experimentally, ASE was tested for pure and doped BABCz films, and no obvious ASE phenomenon was found. The reason was speculated to be due to the perpendicular orientation of anthracycline and styrene in BABCz, which hindered coplanar π-π delocalization formation.

### 3.3. Theoretical Calculations and Electrochemical Properties

**Figure 7:**
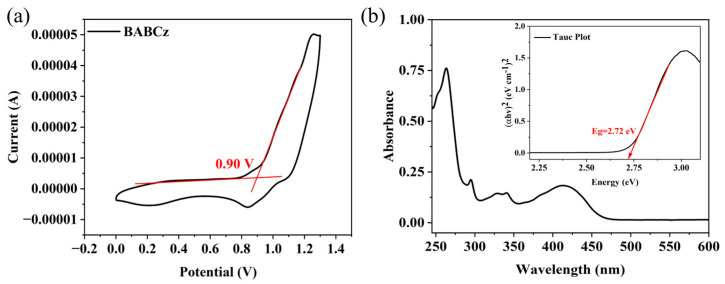
(**a**) Cyclic voltammetry curve of BABCz; (**b**) calculating energy gap by the Tauc plot.

**Figure 8:**
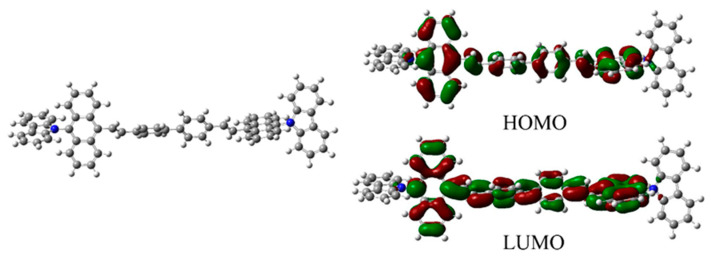
Molecular optimization structure (**left**); Frontier orbital energy level distribution (**right**) of BABCz.

The electrochemical properties of BABCz were investigated by cyclic voltammetry (CV) (Figure 7) and Gaussian simulation (Figure 8). A glassy carbon electrode was used as the working electrode, a platinum electrode as the counter electrode, and Ag/AgNO_3_ as the reference electrode by formulating a 0.1 M solution of tetra-n-butylammonium hexafluorophosphate in dichloromethane as the electrolyte. Ferrocene was added as an internal standard (Fc^+^/Fc corresponds to a vacuum level of 4.8 eV), and the electrolyte was purged with nitrogen to remove oxygen. The BABCz solution was tested at a scanning rate of 0.1 V/s at room temperature. With Ag/AgNO_3_ as the reference electrode, φ(Fc^+^/Fc) is 0.05 V, and φ_Ox_ was measured at 0.90 V (Figure 7a). According to the formula E_HOMO_ = −e (φ_ox_ − φ(Fc^+^/Fc) + 4.8) (eV), the E_HOMO_ was calculated to be equal to −5.65 eV. And the band gaps E_g_ can be obtained through the Tauc plot((αhv)^1/m^ = B (hν − E_g_)) [33], with a value of 2.72 eV (Figure 7b). Finally, the LUMO energy level is obtained to be −2.93 eV according to E_LUMO_ = E_HOMO_ − E_g_.

The anthracene is nearly perpendicular to the intramolecular carbazole and styrene building blocks, making the conjugated planar structure shorter, which was also confirmed by the single-crystal structure. The electron clouds of the HOMO and LUMO orbitals of this molecule are equally distributed over the anthracyl and styrene building blocks, with little distribution for carbazoles. According to the theoretical calculation, the HOMO energy level is −5.21 eV and the LUMO energy level is −2.20 eV.

The experimentally measured values by the CV method were −5.65 eV and −2.93 eV, respectively. A band gap of 2.72 eV. The HOMO and LUMO energy levels obtained by theoretical calculations are also summarized in Table 2.

### 3.4. Device Fabrication and Performance

**Figure 9:**
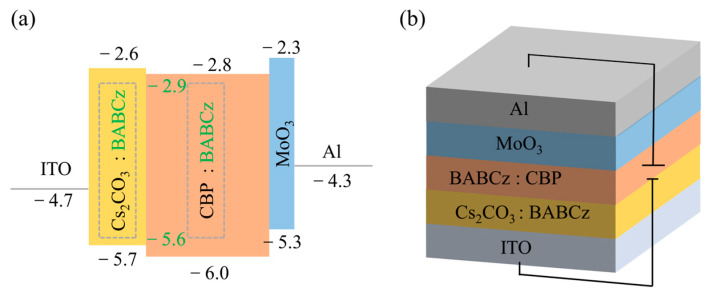
(**a**) Energy level diagram of the OLED device; (**b**) OLED device structure.

**Figure 10:**
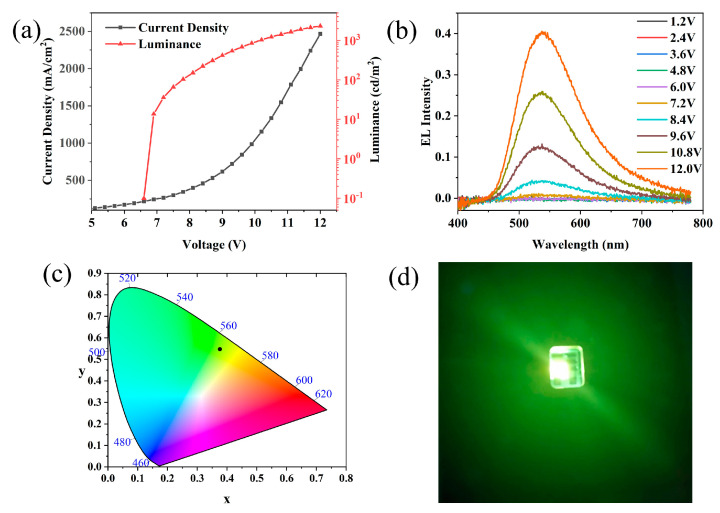
(**a**) J-V-L characteristic curve; (**b**) spectral curve under different voltages; (**c**) chromaticity coordinate; (**d**) OLED device based on BABCz.

To examine the electroluminescence capability of BABCz, a simple three-layer structure of the inverted OLED device was fabricated. A mixed layer of cesium carbonate and BABCz was used as the electron transport layer, a mixed layer of CBP and BABCz as the luminescence layer, and molybdenium oxide as the hole transport layer. Mixed material with BABCz is used in the electron transfer layer to reduce the barrier between the luminous layer and doping with CBP is used to improve the film quality and luminous efficiency. The device structures are ITO/Cs_2_CO_3_:BABCz (1:6) (35 nm)/CBP:BABCz (5:1) (100 nm)/MoO_3_ (5 nm)/Al (250 nm). (Figure 9). The results show that the simple OLED device based on BABCz can emit light normally at voltages of 6.6–12 V (Figure 10). The emission peak of the device is 535 nm, showing blue-green light, and the maximum brightness can reach 2300 cd/m^2^. This indicates that BABCz films have electroluminescence properties.

## 4. Conclusions

In this work, a novel anthracene-based emitting material, BABCz, was designed and synthesized. The introduction of anthracene, despite weakening the luminescence ability of single molecules, also avoids the fluorescence quenching resulting from intermolecular aggregation in the solid state. BABCz has good thermal stability and electroluminescent properties and can be doped with CBP to prepare highly uniform and flat films. The simplest three-layer OLED device with this material as the luminescent layer can be illuminated normally in the range of 6.6–12 V, indicating the potential of this material for OLED fabrication.

## Figures and Tables

**Table 1:** Photophysical parameters of BABCz and BSBCz.

Materials	λ_PL_ (nm)	Φ_PL_ ^(a)^	τ (ns)	k_r_/10^8^(s^−1^) ^(c)^
BABCz(CHCl_3_ solution)	523	8.93%	1.42	0.63
BSBCz(CHCl_3_ solution)	423, 443	78.6%	1.06	7.42
BABCz(vacuum-deposited pure film) ^(b)^	518	-	-	-
BSBCz(vacuum-deposited pure film)	457, 481	70.2%	1.09	6.44
BABCz(vacuum-deposited doped film)	507	23.33%	1.76	1.33

(a) Photoluminescence quantum yields (Φ_PL_) were measured by integrating sphere. (b) No observable value was measured. (c) k_r_ = Φ_PL_/τ.

**Table 2:** HOMO and LUMO data comparison.

Method		HOMO (eV)	LUMO (eV)	Band Gap (eV)
BABCz	CV	−5.65	−2.93	2.72
	Calculation	−5.21	−2.20	3.01

## Data Availability

The datasets used and analyzed in the current study are available from the corresponding author upon reasonable request.

## Supplementary Materials

The following supporting information can be downloaded at: https://www.mdpi.com/article/10.3390/ma16103685/s1. NMR and MS spectra.
