# Supplementary material for: An Anthracene-Based Bis-Stilbene Derivative as Luminescent Materials for Organic Light Emitting Diodes
Source: Materials (Basel). 2023 May 12;16(10):3685. doi: 10.3390/ma16103685 (PMC10221538; doi:10.3390/ma16103685)
Supplement: Supplementary file 1 [file materials-16-03685-s001.zip › materials-2370147-supplementary.pdf]

# An Anthracene-Based Bis-Stilbene Derivative as Luminescent Materials for Organic Light Emitting Diodes

**Hui Wang [1,2,3,4], Houlin Wu [3], Guangling Bian [2,3,4,*] and Ling Song [3,4,*]**

1 College of Chemistry and Materials Science, Fujian Normal University, Fuzhou 350007, China; wanghui@fjirsm.ac.cn
2 Fujian Science & Technology Innovation Laboratory for Optoelectronic Information of China, Fuzhou 350108, China
3 The Key Laboratory of Coal to Ethylene Glycol and Its Related Technology, Fujian Institute of Research on the Structure of Matter, Chinese Academy of Sciences, Fuzhou 350002, China; wuhoulin@fjirsm.ac.cn
4 Fujian College, University of Chinese Academy of Sciences, Fuzhou 350002, China
* Correspondence: glb@fjirsm.ac.cn (G.B.); songling@fjirsm.ac.cn (L.S.)

**Figure S1.** The $^{1}H$ NMR spectra of 10-bromoanthracene-9-carbaldehyde

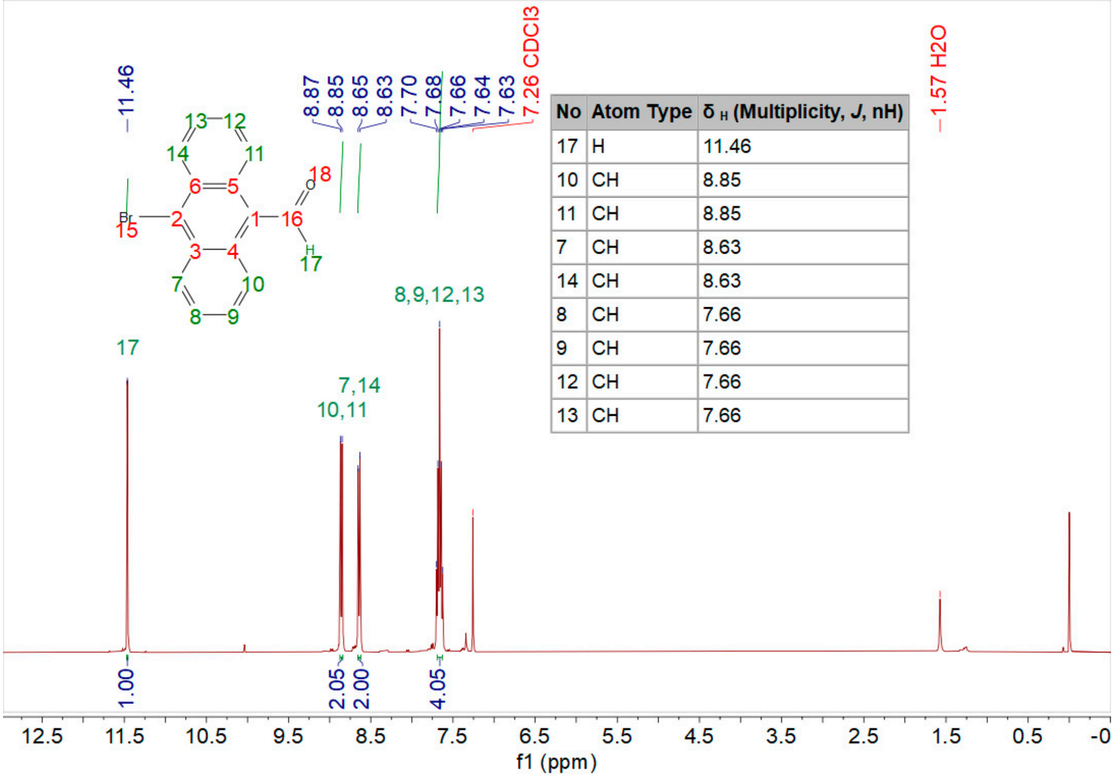


| No | Atom Type | δ H (Multiplicity, *J*, nH) |
|---|---|---|
| 17 | H | 11.46 |
| 10 | CH | 8.85 |
| 11 | CH | 8.85 |
| 7 | CH | 8.63 |
| 14 | CH | 8.63 |
| 8 | CH | 7.66 |
| 9 | CH | 7.66 |
| 12 | CH | 7.66 |
| 13 | CH | 7.66 |

**Figure S2.** The $^{13}C$ NMR spectra of 10-bromoanthracene-9-carbaldehyde

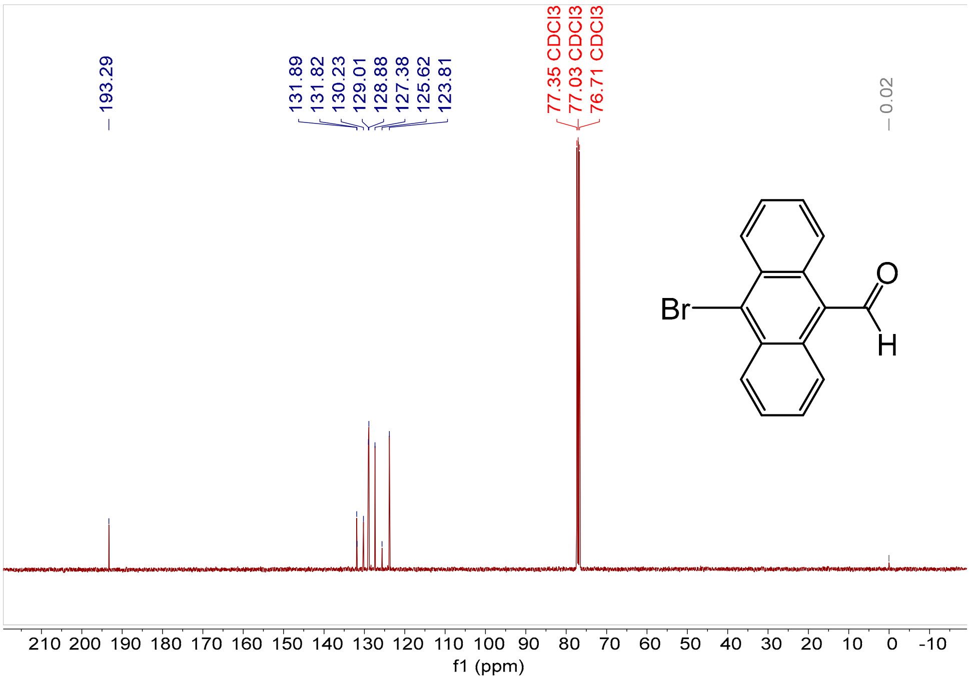

**Figure S3.** The ESI-TOF-MS spectra of 10-bromoanthracene-9-carbaldehyde（Positive Ion Mode）

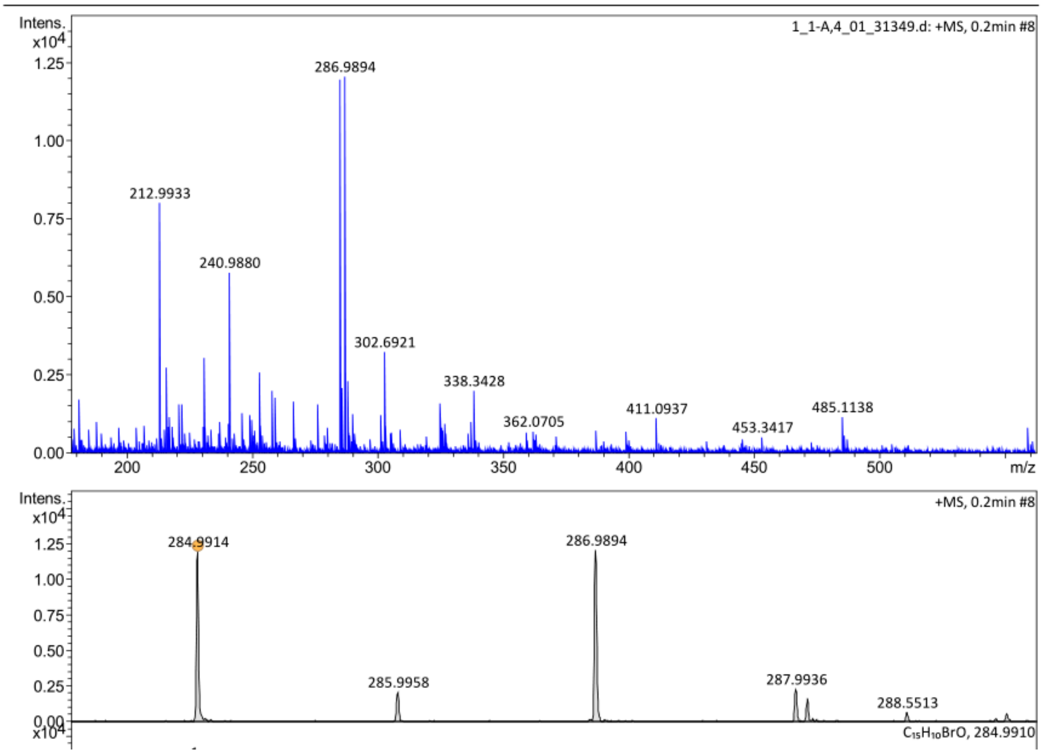


**Figure S4.** The $^{1}$H NMR spectra of 10-(9H-carbazol-9-yl)anthracene-9-carbaldehyde

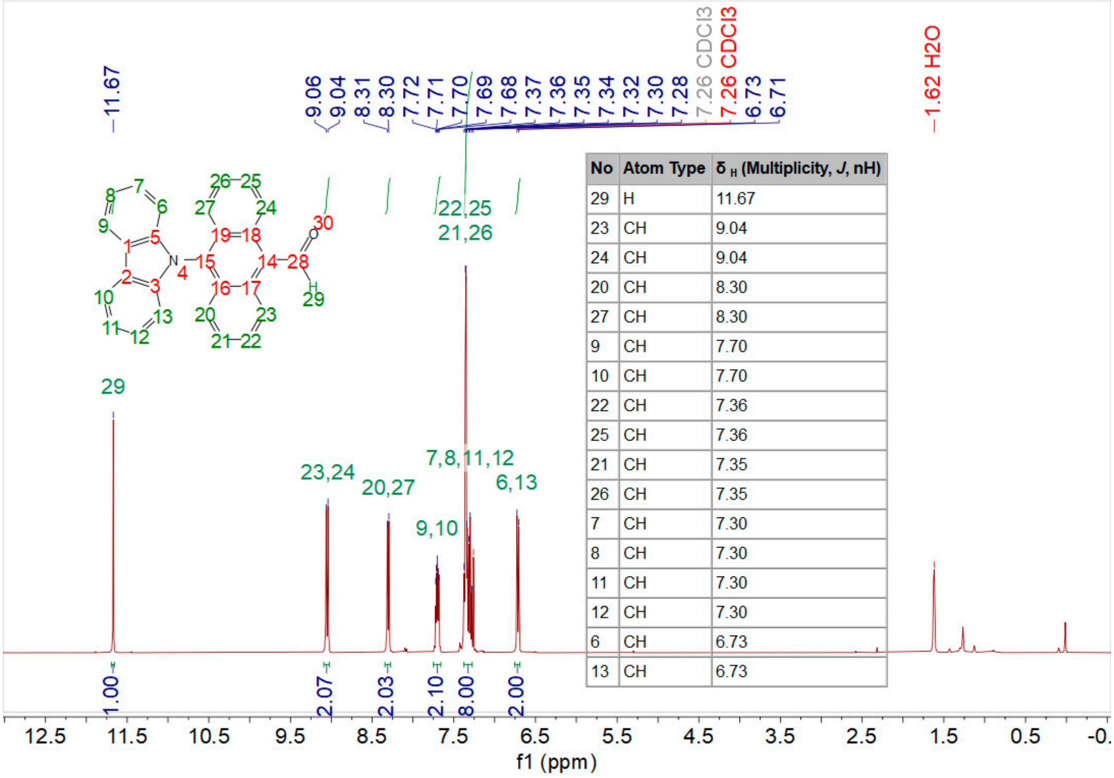


| No | Atom Type | δ H (Multiplicity, J, nH) |
|---|---|---|
| 29 | H | 11.67 |
| 23 | CH | 9.04 |
| 24 | CH | 9.04 |
| 20 | CH | 8.30 |
| 27 | CH | 8.30 |
| 9 | CH | 7.70 |
| 10 | CH | 7.70 |
| 22 | CH | 7.36 |
| 25 | CH | 7.36 |
| 21 | CH | 7.35 |
| 26 | CH | 7.35 |
| 7 | CH | 7.30 |
| 8 | CH | 7.30 |
| 11 | CH | 7.30 |
| 12 | CH | 7.30 |
| 6 | CH | 6.73 |
| 13 | CH | 6.73 |

**Figure S5.** The $^{13}$C NMR spectra of 10-(9H-carbazol-9-yl)anthracene-9-carbaldehyde

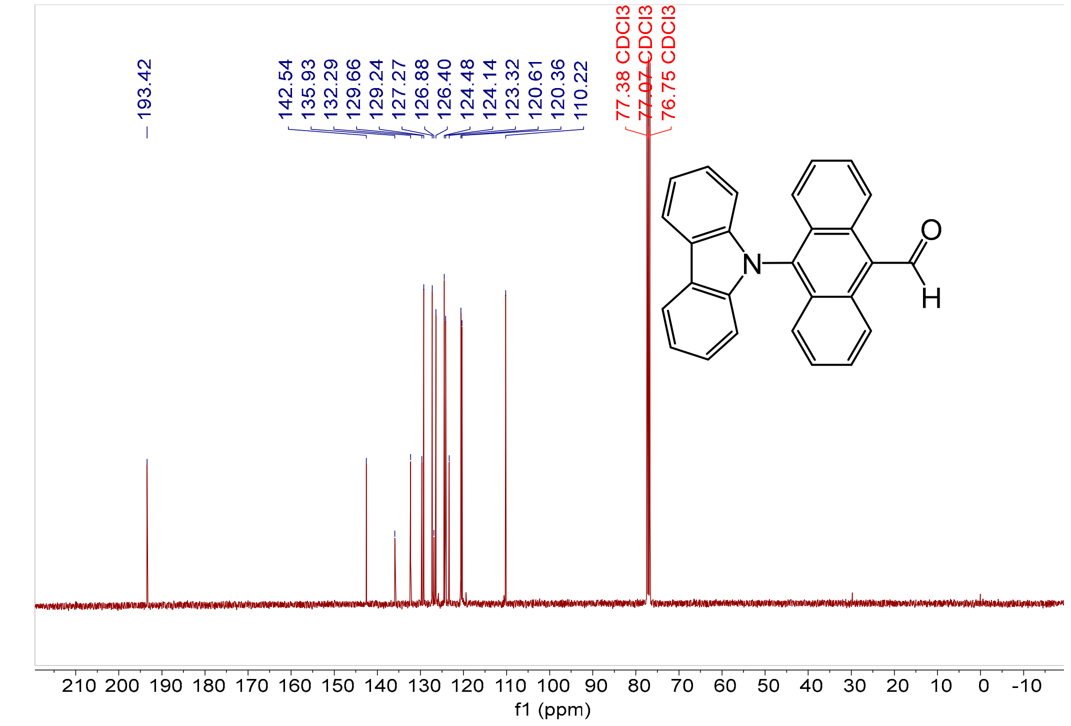


**Figure S6**. The ESI-TOF-MS spectra of 10-(9H-carbazol-9-yl)anthracene-9-carbaldehyde（Positive Ion Mode）

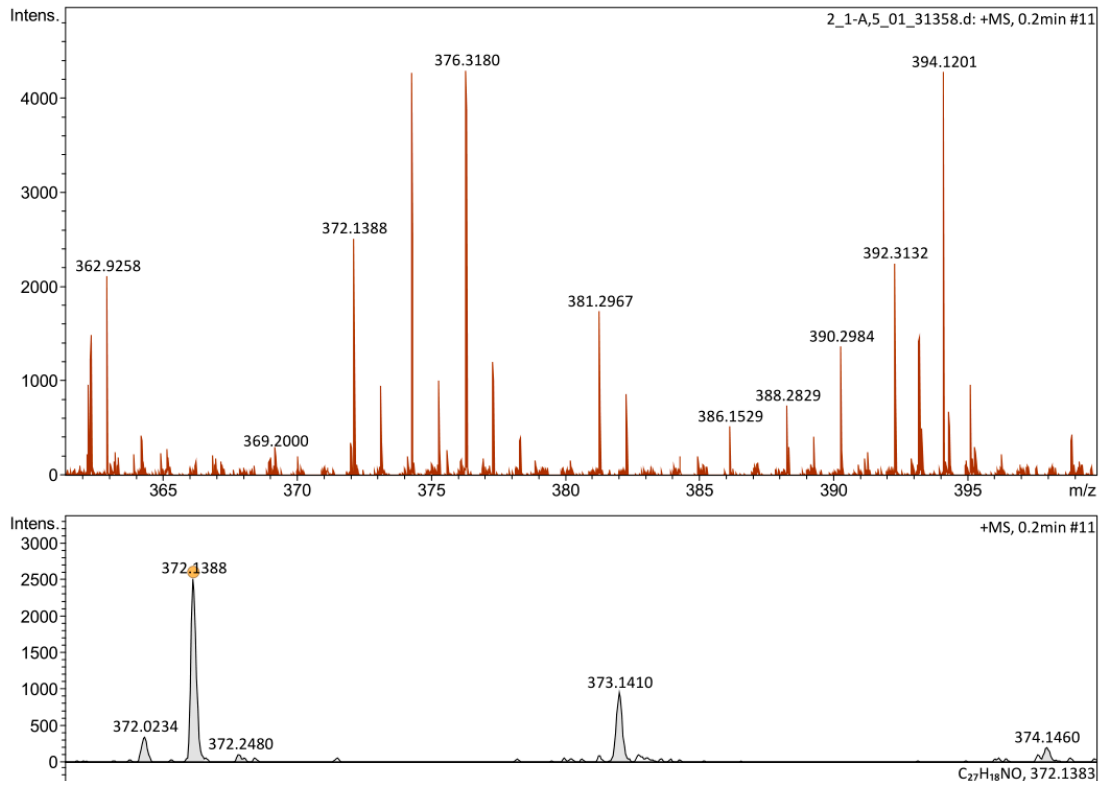

**Figure S7.** The $^{1}$H NMR spectra of BABCz

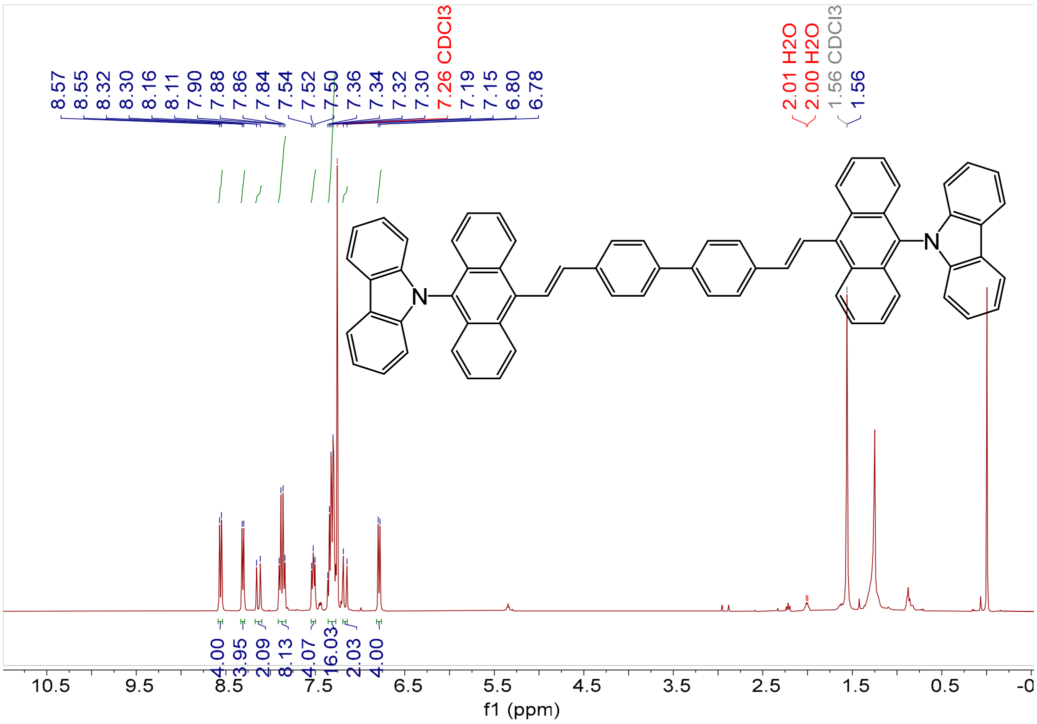


**Figure S8.** The MALDI-TOF-MS spectra of BABCz（Positive Ion Mode）

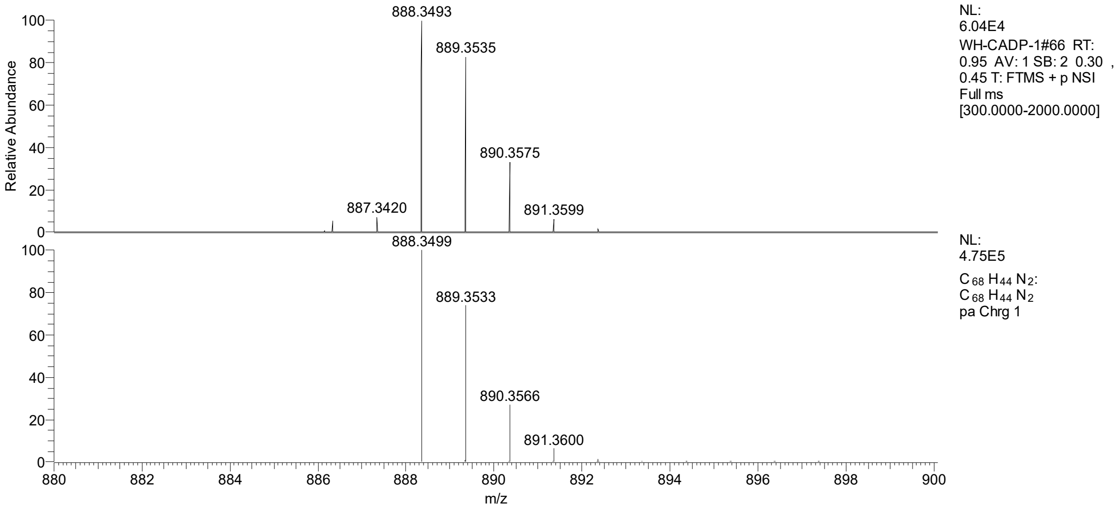


Elemental composition search on mass 888.3493

m/z= 883.3493-893.3493

| m/z | Theo. Mass | Delta (ppm) | RDB equiv. | Composition |
|---|---|---|---|---|
| 888.3493 | 888.3499 | -0.70 | 48.0 | $C_{68}H_{44}N_2$ |
